# Getah Virus Infection Rapidly Causes Testicular Damage and Decreases Sperm Quality in Male Mice

**DOI:** 10.3389/fvets.2022.883607

**Published:** 2022-04-25

**Authors:** Fengqing Li, Bing Zhang, Zhiwen Xu, Chaoyuan Jiang, Mincai Nei, Lei Xu, Jun Zhao, Huidan Deng, Xiangang Sun, Yuancheng Zhou, Ling Zhu

**Affiliations:** ^1^College of Veterinary Medicine, Sichuan Agricultural University, Chengdu, China; ^2^College of Animal Science, Xichang University, Xichang, China; ^3^Sichuan Techlex Food Co., Ltd., Mianyang, China; ^4^Key Laboratory of Animal Diseases and Human Health of Sichuan Province, Chengdu, China; ^5^Livestock and Poultry Biological Products Key Laboratory of Sichuan Province, Sichuan Animal Science Academy, Chengdu, China; ^6^Animal Breeding and Genetics Key Laboratory of Sichuan Province, Sichuan Animal Science Academy, Chengdu, China

**Keywords:** Getah virus, male mice, damage, testicular, sperm

## Abstract

Getah virus (GETV) is a zoonotic arbovirus that can cause infection in many animals. It can cause pyrexia and reproductive losses in animals. The objective of the study was to explore the effects of GETV on male reproductive ability. Male mice were injected with 100 × TCID_50_/0.1 ml in a volume of 100-μL GETV in their hindquarter muscle, resulting in decreased semen quality and testicular histopathological changes, and the virus was detected in the testes. At 0.5 dpi (day post-infection), male mice showed decreased sperm density, motility, and decreased serum testosterone concentration, an increased sperm malformation rate, vacuoles in spermatogonial cells/spermatocytes in spermatogenic tubules, and the highest virus copies in testis. At 2 dpi, the sperm density and motility reached the lowest value of 3.99 × 10^6^/ml and 62.03%, and the malformation rate reached 43.67%. At 28 dpi, the sperm indexes of the experimental group gradually approached that of the control group, but there were still significant differences. Since then, histopathological changes have worsened, with the most severe histopathological changes at 7 dpi and gradual recovery. Up to 14 dpi, the virus was detected by qRT-PCR and immunohistochemistry, which showed that the virus was only present in the testicular interstitium. GETV infection can rapidly enter the testis of mice and reduce the semen quality of mice, which needs to be paid attention to in the prevention and control of GETV.

## Introduction

Getah virus is a spherical particle with about 70 nanometers and a symmetrical 20-hedral body. It belongs to the genus *Alphavirus*, a member of the family *Togaviridae*, and belongs to the *Semliki* Forest virus complex (1).

GETV has been reported in more than a dozen countries and regions worldwide since it was first isolated from Culex mosquitoes captured in Malaysia in 1955 (2). At present, the research on the Getah virus mainly focuses on detecting GETV and virus isolation from mosquitoes and the serological investigation of different animals, including pigs, sheep, and ducks. To date, GETV infection has been detected in various animals distributed in China (3, 4). In 1964, the GETV strain of mosquito type M1 was first isolated in Hainan Province (5), and then mosquito-borne GETV strains were found in Yunnan (6), Sichuan (4), Guizhou, Gansu (1), Jilin, Hebei, and Shanxi provinces. GETV infection in Chinese pigs was first detected in Taiwan in 2002, followed by Henan (2005), Hunan (2017), Anhui (2017), Sichuan (2018), and other provinces (7). GETV infection occurred in Shandong blue foxes in 2017, which may be related to products from pigs infected with the geta virus. GETV was found in febrile cattle in Jilin Province in 2018, and the positive rate of bovine serum was 83.3% (8).

GETV can cause clinical manifestations in a variety of animals. Rashes, hind limb edema, fever, and lymph node enlargement were observed in infected horses (9). Piglets infected with GETV showed depression, tremor, hind limb paralysis, diarrhea, and high mortality (10). Histopathological examination showed that GETV-infected pigs had neurodegenerative changes (neuronophagocytosis and central chromatin dissolution), pneumocytes detachment, renal cortical thickening, hemorrhagic splenitis (11). Infected sows had abortions (12).

The occurrence of abortion is related to female animals and closely related to the semen quality of male animals (13, 14). The reproductive effects of GETV on male animals have not been reported (15, 16). Therefore, we explored the effects of the Getah virus on testis, epididymis, and sperm in mice to reveal the reproductive effects of GETV in male mice.

## Materials and Methods

### Animals and Treatment

The study was carried out on adult male specific-pathogen-free mice with body weights of 35–40 g obtained from Chengdu Dossy Experimental Animals CO., LTD. Permission for the use of animals was obtained from Sichuan Agricultural University Animal Ethical and Welfare Committee. All experiments followed relevant regulations and standards. The mice were allowed to adapt for about 1 week prior to the commencement of the experiment. The mice were kept under standard conditions, including inverted 12-h light/dark cycles, constant temperature (22 ± 2°C), and humidity (70 ± 4%) with access to standard feed and water *ad libitum*. After acclimatization, the mice were randomly assigned to groups.

Experimental groups: the male mice were intramuscularly injected with 100 × TCID_50_/0.1 ml in a volume of 100-μL GETV at the posterior limb. At 0.5, 1, 2, 3, 5, 7, 14, 21, 28 dpi, the mice were euthanized. Then, the testis, epididymis, and serum were harvested for further analysis (10 mice each group).

The control group: The mice were intramuscularly injected with 100-μL saline at posterior limb as mock control. Other treatments were consistent with the experimental group.

Virus: The GETV was isolated from aborted sows and stored in Animal Biology Technology Center. The getah virus was cultured and titrated with BHK21 cells *in vitro*.

### Epididymal Sperm Analysis

The right cauda part of epididymis was isolated immediately, thoroughly minced in 1 ml of a sperm cell BWW medium (Leagene Biotechnology, Inc., Cat No.: CM0052) at 37°C and thermostatically incubated for 0.5 h to obtain sperm suspension and used for the determination of a sperm count, sperm motility, and sperm morphology.

The total number of sperms was calculated by CASA using the Sperm Class Analyzer, and the count was expressed in millions/ml. Total motility (%) was recorded.

10 μl of sperm suspension was dropped on a slide to make a thin smear. The smear was dried at 37°C for 5 min (3 smears for each sample). The smears were fixed and stained; spermatozoa microscope images were captured by oil immersion. The abnormal sperm of 100 spermatozoa was counted at 3 different areas per smear and expressed in percentage, specifying the most common defects (17).

The cauda-epididymidis was fixed with 2.5% glutaraldehyde and then sent to the Lilai biomedicine experiment center (Chengdu, Sichuan) for electron microscopy to observe the ultrastructure of sperm in the epididymis.

### Organ Indices and Evaluation of Histology

At the appointed time, 10 mice in the control group and 10 in the experimental group were anesthetized with ether. The weights were measured, and the blood was collected into tubes by eye enucleation. The mice were humanely sacrificed by cervical dislocation. Testis and epididymis were isolated. We removed other tissue that adhered to the testis and blotted it dry with blot paper. After blotting, the isolated testis was weighed to the nearest milligram. Tissue somatic indices were determined using the formula:


(1) $$\begin{array}{r} TSI=\frac{\text{Weight of the tissue}}{\text{Weight of the mice}}\times 100\% \end{array}$$

The right caudal epididymis was harvested for sperm analyses. The left testes and epididymis were fixed in 2% paraformaldehyde-2.5% glutaraldehyde solution. Three were sectioned and stained routinely with hematoxylin and eosin, and then viewed under a light microscope for histopathological examination. The other three testes and epididymis were sent to the Lilai biomedicine experiment center (Chengdu, Sichuan) for electron microscopy to observe the ultrastructure of cells in the testis.

### Estimation of Testosterone Concentrations

According to the manufacturer's instructions, the free testosterone level in serums was quantified by the ELISA kit (Abcam, USA). The absorbance was recorded at 450 nm. The minimum detection limit in the testosterone assay was.1 ng/ml. The cross-reactivity assayed with other steroids tested showed 0.01%.

### Detection of Getah Virus in Testis

The harvested right testis was weighed and grounded in liquid nitrogen. All grounded tissues from the infected mice were stored at −80°C until virus titration. Viral RNAs were extracted with a universal RNA kit [Cofitt Life Sciences (HK) Limited] and determined by real-time qRT-PCR on a Roche LightCycler® 96 using TB Green Premix Ex Taq ^TM^II (TaKaRa, Japan). After comparison with a standard curve, viral load in testicles was expressed as viral RNA copies per μg RNA. The primer sets for these assays are described (Table 1).

**Table 1:** Fluorescence quantitative primer information.

**The primer name**	**The primer sequence (5^**′**^-3^**′**^)**	**The length (bp)**
Cap^−^ F	CTTGACGGTAAGGTCACGGG	104
Cap^−^ R	GTAAGCTTCGCTAGGTCGGG	

The paraffin blocks of tissues were used for an immunohistochemical assay by the method of Wenqiang Ma and Wanpeng Yu (18, 19) by a SABC immunohistochemical staining kit (BOSTER, SA1020). Deparaffinization, rehydration, and antigen retrieval were performed on the 5-mm paraffin sections of the testis or epididymis. The tissue sections were treated with 3% H2O2 in PBS (pH7.6) for 10 min and 5% BSA for 30 min. And then we incubated the sections at 4°C overnight with a Rabbit antibody against the E2 protein of the Getah virus as primary antibodies. The sections were rinsed with PBS three times and incubated with biotin-labeled goat anti-rabbit as secondary antibodies for 30 min. After incubation with SABC for 30 min, the specific binding on the sections was visualized by DAB. The sections were counterstained with hematoxylin.

## Results

### Virus Infection Affects Sperm Parameters

**Figure 1:**
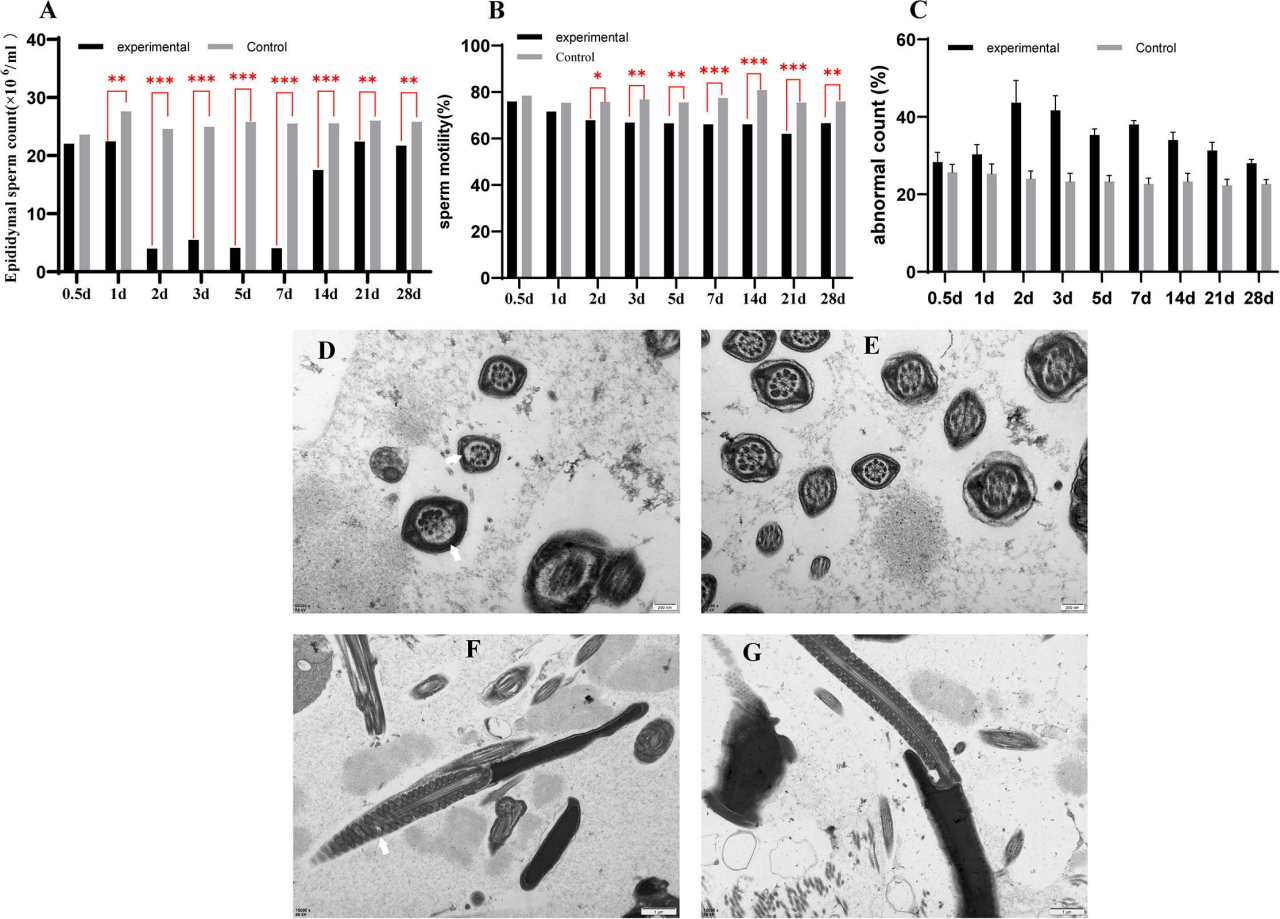
The sperm count of epididymal **(A)**, the sperm motility of epididymal **(B)**, the abnormal sperm count of epididymal **(C)**. A transmission electron microscope photograph of sperm in epididymal **(D–G)**. **(D,F)** are transmission electron micrographs of sperm flagellum in a GETV-infected mouse; **(E,G)** are transmission electron micrographs of sperm flagellum in the control mouse; the scale of **(D,E)** is 200 nm; the scale of **(F,G)** is 1 μm. Partial peripheral microtubules disappear (a thin white arrow); swollen mitochondria in sperm midpiece (a thick white arrow).

We examined the sperm count, sperm motility, and the abnormal sperm count in epididymal of normal and virus-infection mice. The sperm count of the virus-infection mice decreased to its lowest at 2 dpi, at 3.99 × 10^6^/ml, while it was 24.57 × 10^6^/ml in the control group. Sperm counts of the experimental group were at a low level until 7 dpi but increased to 17.53 × 10^6^/ml at 14 dpi. In addition, all had significant decreases in the experimental group from 1 to 28 dpi (Figure 1A). Sperm motility was also lower in the experimental mice than in the control mice (Figure 1B), significant during 1 dpi to 28 dpi. The lowest sperm motility was 62.03% at 21 dpi. Similarly, the experimental group had more teratospermia, and the rate of abnormality was the highest at 2 dpi (43.67%) and was significantly higher than the control group except at 0.5 dpi (Figure 1C).

The epididymis was examined by electron microscopy, and the image showed that the axoneme in spermatozoa of experimental mice was missing in different degrees (Figure 1D), and the mitochondria were swollen (Figure 1F). There were no abnormalities in the control group (Figures 1E,G).

### The Testis Is Histopathologically Altered by Getah Virus Infection

**Figure 2:**
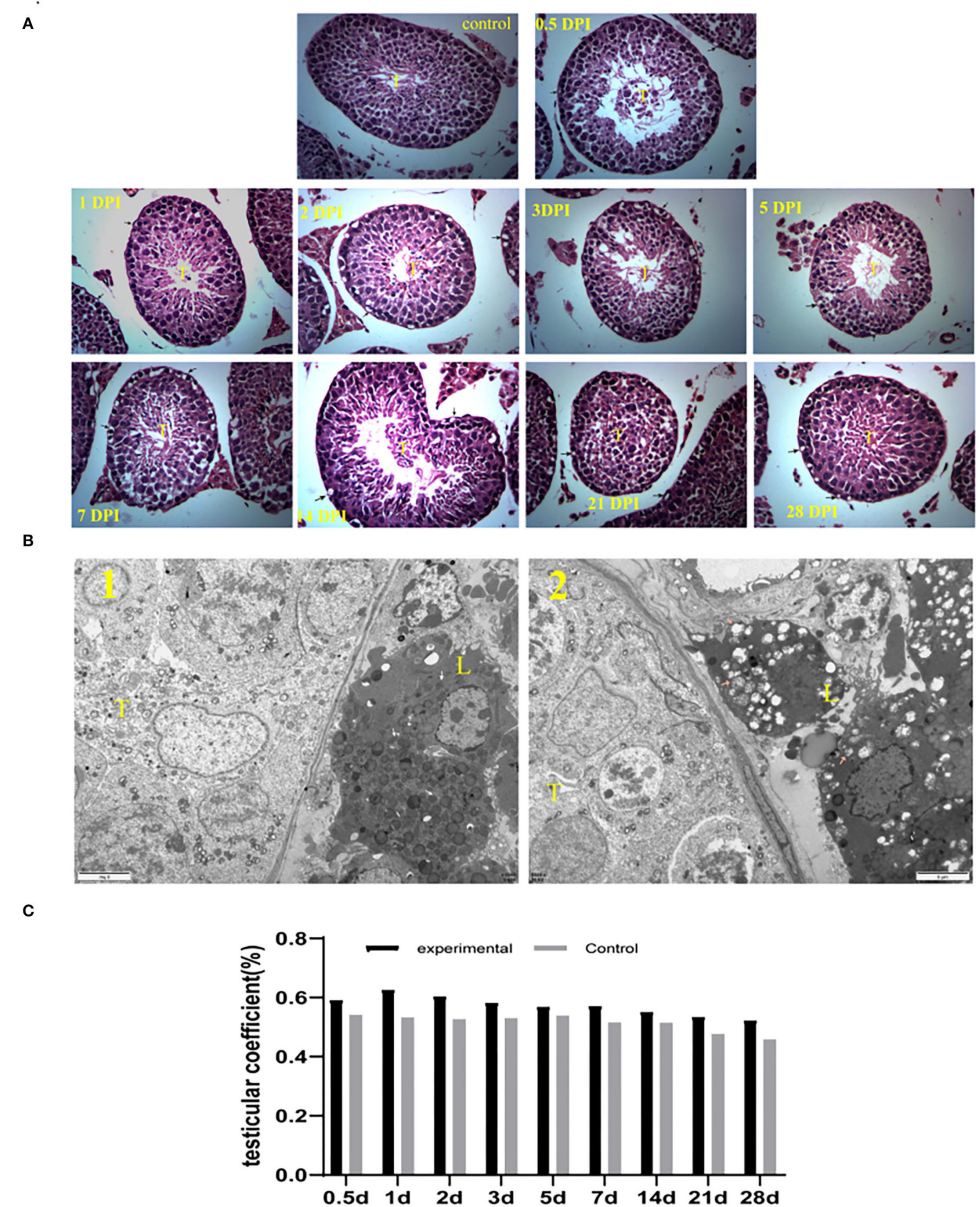
**(A)** Histological (HandE) staining of testes tissue sections (40 × 10). (control) Control testes from the control mice were given an injection with PBS at 7 dpi. The other is GETV-infected testes at 0.5 dpi-28 dpi. cavitation in spermatogonia and spermatocyte (arrows), T indicates lumens of seminiferous tubules. **(B)** The testis's transmission electron microscope photograph 1: the control mouse; 2: the GETV-infected mouse. The pink arrow in B: normal mitochondria in Leydig cells. The pink arrow in B: Swollen mitochondria in Leydig cells. **(C)** Changes in testicular coefficient after infection of the GETV mice over time.

Testes tissue morphology was observed under the light microscopy after H&E staining. The testicular tissue of the experimental mice appeared to have severe cavitation, irregular with wide spaces in seminiferous tubules, which decreased spermatogonia and spermatocytes. Moreover, sperm in lumens of seminiferous tubules decreased in GETV-treated groups at all appointed times (Figure 2A). In the 1st week, the pathological damage of the testis gradually increased and began to improve on the 14th day. On the 28th day, the cavitation was reduced and arranged regularly, and the sperm cells appeared in the center of the seminiferous tubules.

The transmission electron microscope photographs showed that many mitochondria were swollen in mouse Leydig cells in the experiment group (Figure 2B).

However, no significant difference was found in the testicular coefficient between the mice of the experimental group and the control group (*p* >0.05) (Figure 2C).

### GETV Infection Reduces Testosterone Synthesis in Male Mice

**Figure 3:**
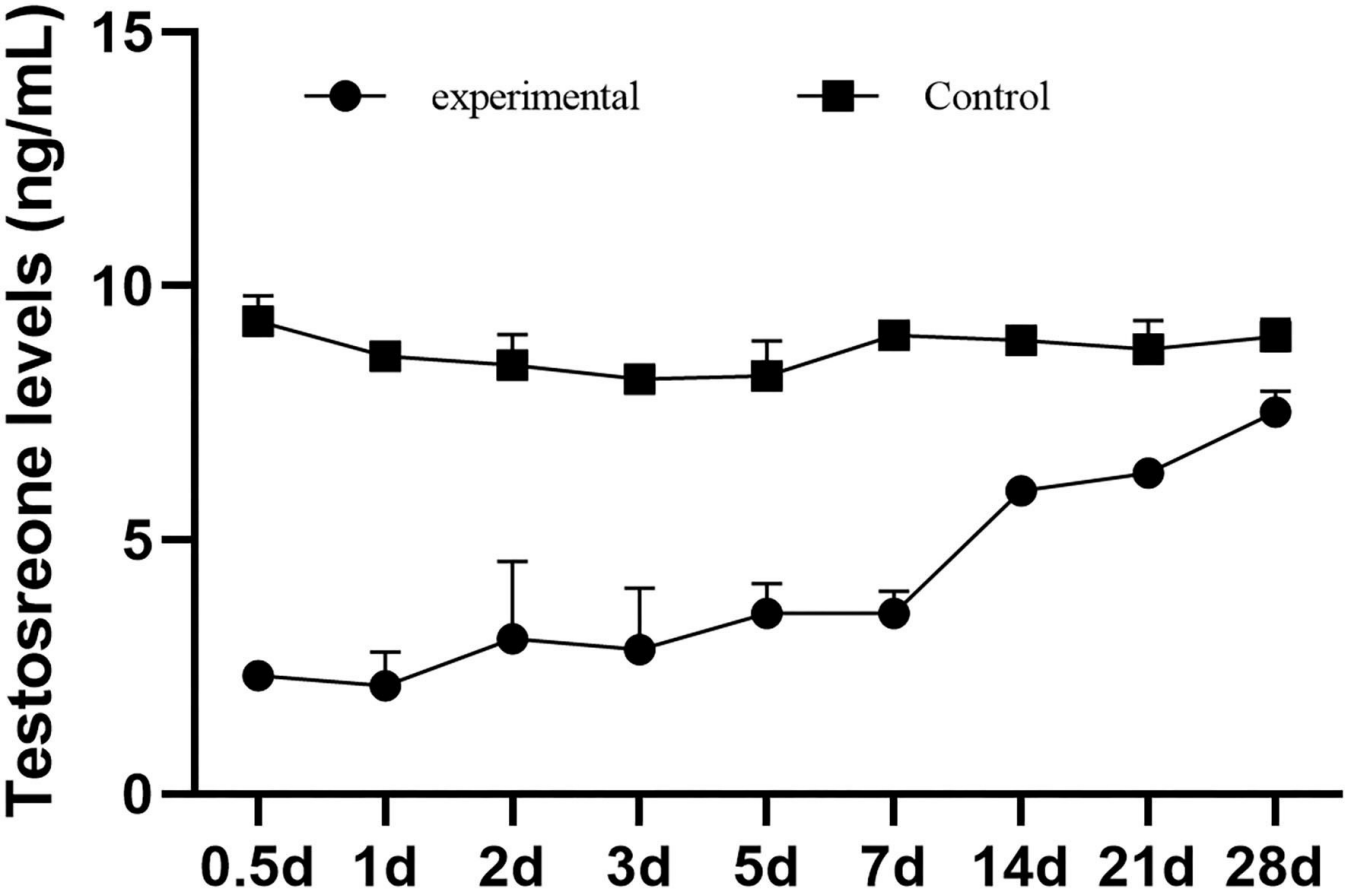
The testosteron levels of male mice in serum.

The testosterone concentrations of the male mice in the control group were consistently stable and significantly higher than those in the experimental group. The average testosterone concentration in the control group was 8.72 ng/ml, and that in the experimental group was 4.14 ng/ml. After infection, the testosterone level of the male mice decreased sharply, which dropped to 2.33 ng/ml at 0.5 dpi and reached the lowest level of 2.14 ng/ml at 1 dpi. Then, it gradually increased to 7.52 ng/ml, which was closest to the control group at 28 dpi (Figure 3).

### GETV Was Detected in Testis of Mice

**Figure 4:**
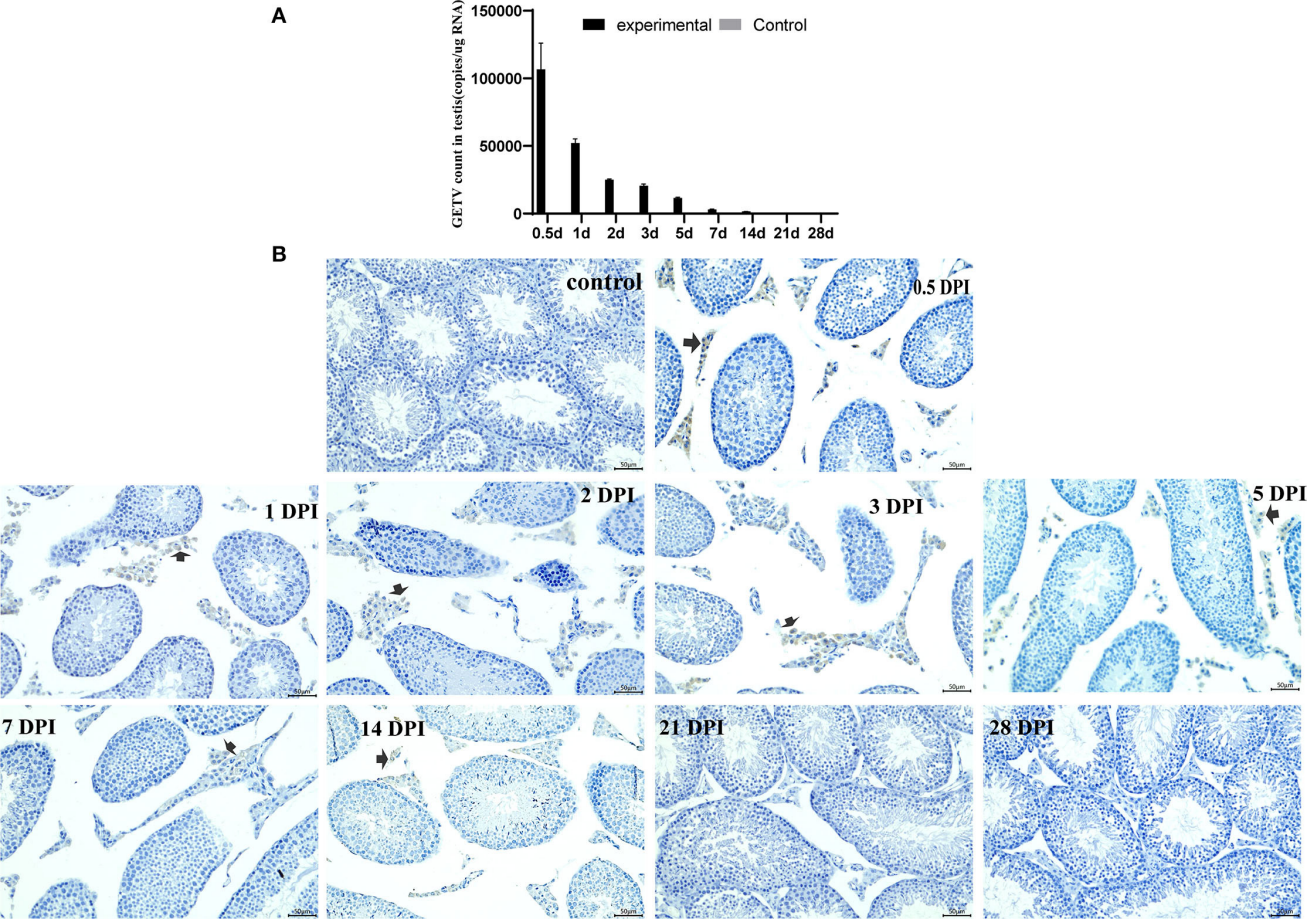
Detection and persistence of Getah virus in the testis. **(A)** The GETV copies in the testis; **(B)** the testis immunohistochemical images. (control) Control testes from the control mice were given an injection with PBS at 7 dpi. The other is GETV-infected testes at 0.5–28 dpi. GETV E2 protein-positive cells are brown (arrows).

The virus in testes was detected by real-time qRT-PCR and the immunohistochemical assay. Viral RNA copies per ug RNA were used to indicate the virus in the testes. The highest viral content in the testes of the experimental mice was 1.07 × 10^5^ copies/ug RNA at 0.5 dpi, and the virus disappeared at 21 dpi. GETV was not detected in the control group mice (Figure 4A).

The virus was observed in the Leydig only at the testis immunohistochemical images of the experimental mice. Many GETV E2 positive cells appeared in the Leydig at 0.5 dpi, and then the positive cells decreased gradually and disappeared at 21 dpi (Figure 4B).

## Discussion

The male reproductive system has an immunosuppressive environment to protect the spermatozoon from detrimental immune responses (20). A broad range of virus families has been detected in the male reproductive system, impairing male fertility and leading to sexual transmission of viruses (21). The testis is an immune-privileged organ with restricted drugs penetration, considered to constitute a tissue reservoir for emerging viruses, such as Zika and Ebola (22). In this study, we demonstrated that GETV could exist in the testis and distribute in the Leydig of testicular, which caused testicular damage and decreased sperm quality in the mice.

The male mice infected with GETV showed a decrease in sperm quality, which was manifested as a decrease in sperm density and viability, and an increase in the malformation rate. Therefore, it is believed that GETV infection will cause reduced fertility in the male mice (23). Only 0.5 days after infection, the virus appeared in the testicular Leydig and caused testicular histopathological changes and decreased semen quality and testosterone levels. It suggested that there is a mechanism for virus to quickly penetrate through the blood-testosterone barrier, which can be discussed in subsequent studies.

Testosterone, as one of the most important androgens in males, is mainly synthesized by Leydig cells from cholesterol. It plays an important role in spermatogenesis (24). After viral infection, the sperm count of the mice decreased and remained at a low level during 2 dpi to 7 dpi, and significantly increased at 14 dpi. That is consistent with the change trend of testosterone. Therefore, it is reasonable to speculate that there is a strong relationship between the change in testosterone concentration caused by virus infection and the semen quality of mice. Meanwhile, cavitation in spermatogonia and spermatocyte increased until 7 dpi, and histology gradually returned to normal at 14 dpi, but a small number of vacuoles were still present at 28 dpi, which may associate with changes in testosterone levels too. After GETV infection in the mice, many viruses were detected in testicular tissue, and a large number of E2 protein-positive cells appeared in testicular interstitial at 0.5 dpi, accompanied by a decrease in serum testosterone concentration. Subsequently, the E2-positive cells were less and less in the testicular interstitium, and the serum testosterone concentration began to increase gradually after reaching the lowest value at 1 dpi too. This suggests that virus colonization in the testicular Leydig reduced the mice's ability of secreting testosterone. That could be why GETV infection caused testicular damage and decreased semen quality.

## Data Availability Statement

The raw data supporting the conclusions of this article will be made available by the authors, without undue reservation.

## Ethics Statement

The animal study was reviewed and approved by Sichuan Agricultural University Animal Ethical and Welfare Committee.

## Author Contributions

LZ: conceptualization, writing—review and editing, and revised the manuscript. FL and BZ: writing—original draft and revised the manuscript. ZX and CJ: writing—review. MN and LX: software. JZ and HD: validation. XS and YZ: supervision. All authors contributed to the article and approved the submitted version.

## Funding

This research was funded by the Sichuan Province's 14th Five-Year Plan Sichuan Pig Major Science and Technology Project (Grant Number: 2021ZDZX0010), the Key R&D Program in Rural Areas of Sichuan Provincial Department of Science and Technology (Grant Number: 2020YFN0147), the Sichuan Veterinary Medicine and the Drug Innovation Group of China Agricultural Research System (Grant Number: CARS-SVDIP), and the Open Fund of Sichuan Provincial Key Laboratory of Animal and Poultry Biological Products (2021KF002).

## Conflict of Interest

BZ was employed by Sichuan Techlex Food Co., Ltd.

The remaining authors declare that the research was conducted without any commercial or financial relationships that could be construed as a potential conflict of interest.

## Publisher's Note

All claims expressed in this article are solely those of the authors and do not necessarily represent those of their affiliated organizations, or those of the publisher, the editors and the reviewers. Any product that may be evaluated in this article, or claim that may be made by its manufacturer, is not guaranteed or endorsed by the publisher.

## References

[1] Zhai Y Wang H Sun X Fu S Wang H Attoui H Tang Q Liang G. Complete sequence characterization of isolates of Getah virus (genus Alphavirus, family Togaviridae) from China. J General Virol. (2008) 89:1446–56. 10.1099/vir.0.83607-0 18474561

[2] Fang Y Zhang W Xue J Zhang Y. Monitoring mosquito-borne arbovirus in various insect regions in China in 2018. Front Cell Infect Microbiol. (2021) 11:640993. 10.3389/fcimb.2021.640993 33791242 PMC8006455

[3] Ren T Mo Q Wang Y Wang H Nong Z Wang J. Emergence and phylogenetic analysis of a getah virus isolated in Southern China. Front Vet Sci. (2020) 7:552517. 10.3389/fvets.2020.552517 33344520 PMC7744783

[4] Nie M Deng H Zhou Y Sun X Huang Y Zhu L Xu Z. Development of a reverse transcription recombinase-aided amplification assay for detection of Getah virus. Sci Rep. (2021) 11:20060. 10.1038/s41598-021-99734-7 34625631 PMC8501081

[5] Fan N Sun D Cheng R Fu S Zeng L Wu Q. [Isolation and identification of Arbovirus in Hainan province, 2017-2018]. Zhonghua liu xing bing xue za zhi = Zhonghua liuxingbingxue zazhi. (2020) 41:236–43. 10.3760/cma.j.issn.0254-6450.2020.02.018 32164136

[6] Li Y Fu S Guo X Li X Li M Wang L. Serological Survey of Getah Virus in Domestic Animals in Yunnan Province, China. Vector Borne Zoonotic Dis. (2019) 19:59–61. 10.1089/vbz.2018.2273 29957135

[7] Xing C Jiang J Lu Z Mi S He B Tu C. Isolation and characterization of Getah virus from pigs in Guangdong province of China. Transbound Emerg Dis. (2020) 67:2249–53. 10.1111/tbed.13567 32277601

[8] Liu H Zhang X Li L Shi N Sun X Liu Q. First isolation and characterization of Getah virus from cattle in northeastern China. BMC Vet Res. (2019) 15:320. 10.1186/s12917-019-2061-z 31488162 PMC6729113

[9] Lu G Ou J Ji J Ren Z Hu X Wang C. Emergence of Getah Virus Infection in Horse With Fever in China, 2018. Front Microbiol. (2019) 10:1416. 10.3389/fmicb.2019.01416 31281304 PMC6596439

[10] Yang T Li R Hu Y Yang L Zhao D Du L. An outbreak of Getah virus infection among pigs in China, 2017. Transbound Emerg Dis. (2018) 65:632–7. 10.1111/tbed.12867 29575687

[11] Rattanatumhi K Prasertsincharoen N Naimon N Kuwata R Shimoda H Ishijima K. A serological survey and characterization of Getah virus in domestic pigs in Thailand, 2017-2018. Transbound Emerg Dis. (2021) 69:913–8. 10.1111/tbed.14042 33617130

[12] Xia YH Shi ZC Wang XW Li YT Wang Z Chang HT. Development and application of SYBR Green I real-time quantitative reverse transcription PCR assay for detection of swine Getah virus. Mol Cell Probes. (2021) 57:101730. 10.1016/j.mcp.2021.101730 33848593

[13] Yuan M Huang L Leung W Wang M Meng Y Huang Z. Sperm DNA fragmentation valued by SCSA and its correlation with conventional sperm parameters in male partner of recurrent spontaneous abortion couple. Biosci Trends. (2019) 13:152–9. 10.5582/bst.2018.01292 30971639

[14] Melmer D O'Sullivan T Greer A Moser L Ojkic D Friendship R. The impact of porcine reproductive and respiratory syndrome virus (PRRSV) genotypes, established on the basis of ORF-5 nucleotide sequences, on three production parameters in Ontario sow farms. Prevent Vet Med. (2021) 189:105312. 10.1016/j.prevetmed.2021.105312 33676324

[15] Zhang Y Yu J Tan L Wang X Li R Kim D. Complete genetic dissection and cell type-specific replication of old world alphaviruses, getah virus (GETV) and sagiyama virus (SAGV). J Microbiol (Seoul, Korea). (2021) 59:1044–55. 10.1007/s12275-021-1361-8 34570337

[16] Zhou F Wang A Chen L Wang X Cui D Chang H. Isolation and phylogenetic analysis of Getah virus from a commercial modified live vaccine against porcine reproductive and respiratory syndrome virus. Mol Cellular Probes. (2020) 53:101650. 10.1016/j.mcp.2020.101650 32781023

[17] Akomolafe S Oboh G. Walnut leaf extract acts as a fertility agent in male Wistar albino rats - A search for herbal male fertility enhancer. J Complement Integr Med. (2017) 15. 10.1515/jcim-2017-0076 29148981

[18] Ma W Li S Ma S Jia L Zhang F Zhang Y. Zika virus causes testis damage and leads to male infertility in mice. Cell. (2016) 167:1511–24.e1510. 10.1016/j.cell.2016.11.016 27884405

[19] Yu W Zheng H Lin W Tajima A Zhang Y Zhang X. Estrogen promotes Leydig cell engulfment by macrophages in male infertility. J Clin Invest. (2014) 124:2709–21. 10.1172/JCI59901 24762434 PMC4038558

[20] Hedger MP. Immunophysiology and pathology of inflammation in the testis and epididymis. J Androl. (2011) 32:625–40. 10.2164/jandrol.111.012989 21764900 PMC7166903

[21] Liu W Han R Wu H Han D. Viral threat to male fertility. Andrologia. (2018) 50:e13140. 10.1111/and.13140 30569651

[22] Mahe D Matusali G Deleage C Alvarenga R Satie A Pagliuzza A. Potential for Virus Endogenization in Humans through Testicular Germ Cell Infection: the Case of HIV. J Virol. (2020) 94:e01145–20. 10.1128/JVI.01145-20 32999017 PMC7925188

[23] Li J Luo L Diao J Li Y Zhang S Chen L. Male sperm quality and risk of recurrent spontaneous abortion in Chinese couples: a systematic review and meta-analysis. Medicine. (2021) 100:e24828. 10.1097/MD.0000000000024828 33725837 PMC7969329

[24] Goto T Hirabayashi M Watanabe Y Sanbo M Tomita K Inoue N. Testosterone supplementation rescues spermatogenesis and *in vitro* fertilizing ability of sperm in kiss1 knockout mice. Endocrinology. (2020) 161:bqaa092. 10.1210/endocr/bqaa092 32514526

